# Urine oxalate and citrate excretion in patients with kidney stone disease: An ab initio clinical prediction

**DOI:** 10.14814/phy2.14966

**Published:** 2021-08-02

**Authors:** Calyani Ganesan, Alan C. Pao

**Affiliations:** ^1^ Division of Nephrology Department of Medicine Stanford University School of Medicine Palo Alto CA USA; ^2^ Veterans Affairs Palo Alto Health Care System Palo Alto CA USA

Urine excretion of oxalate and citrate represent key determinants for the risk of calcium oxalate kidney stones, the most common type of kidney stone worldwide. Higher urine oxalate or lower urine citrate excretion will increase the risk for kidney stone recurrence. The molecular details underlying the regulation of urine oxalate and citrate excretion have become clearer over the past two decades. For oxalate, the anion transporter SLC26A6 plays a key role in oxalate homeostasis. SLC26A6 is expressed in the apical membrane of epithelial cells of the proximal tubule, pancreatic duct, stomach, and small intestine, and this transporter exchanges chloride for oxalate in brush border membrane vesicles from the proximal tubule and small intestine (Aronson, 2010). SLC26A6 in the small intestine limits net intestinal absorption of oxalate by secreting oxalate: when SLC26A6 is deleted from mice, net intestinal oxalate absorption increases to such an extent that calcium oxalate bladder stones form because of high urine oxalate excretion (Jiang et al., 2006). With respect to citrate, the sodium dicarboxylate co‐transporter NaDC1 helps to determine the level of urine citrate excretion. NaDC1 is expressed in the apical membrane of epithelial cells of the proximal tubule and small intestine. Citrate is freely filtered by the kidney, and citrate is re‐claimed from the tubular fluid by NaDC1 across proximal tubule cells (Pajor, 2014). Deletion of NaDC1 in mice leads to an increase in urinary citrate (Ho et al., 2007). Citrate prevents calcium stones by sequestering ionized calcium into a complex with higher urine solubility than calcium oxalate or phosphate.

Recent evidence has demonstrated that SLC26A6 and NaDC1 physically and functionally interact, at least in oocyte assays and in a transgenic mouse model (Ohana et al., 2013). Ohana et al. (2013) noticed that SLC26A6 knockout mice excrete not only a high level of urine oxalate but also a low level of urine citrate. These investigators tested whether a functional interaction existed between SLC26A6 and NaDC1: when SLC26A6 and NaDC1 were co‐expressed in *Xenopus laevis* oocytes, SLC26A6 inhibited NaDC1, and NaDC1 reciprocally activated SLC26A6. But does a relationship exist between urine oxalate and citrate excretion in humans? For this to be a possibility, one must assume that this relationship between oxalate and citrate, operative in heterologous expression systems and transgenic animals, is recapitulated in humans of different ages, body weights, and genetic backgrounds. This is the question that Prochaska et al. (2021) attempt to answer with human participants and patients with kidney stone disease in this issue of *Physiological Reports*.

In this study, the investigators hypothesize that the relationship between SLC26A6 and NaDC1 is present in humans and that this oxalate–citrate mechanism is disrupted in stone formers (Prochaska et al., 2021). To test this, the investigators examined 24‐h urine citrate and oxalate excretion in 143 non‐stone formers and 13,155 stone formers from three different data sets: (1) non‐stone formers from University of Chicago kidney stone registry; (2) stone formers from University of Chicago Evaluation and Treatment Program; and (3) stone formers from the Litholink testing service. After adjusting for sex and GI anion absorption, the investigators found that the multivariable regression slope between 24‐h urine citrate and oxalate (normalized to urine creatinine) in non‐stone formers was 3.0 (1.5–4.6; units as Δ urine citrate mmol/creatinine per Δ urine oxalate mmol/creatinine); in contrast, the regression slope between these same parameters in stone formers was only 0.3 (0.2–0.4). When all participants were considered together in a single model, the investigators found that the magnitude of the association between urine citrate and oxalate excretion was lower by 2.8 for stone formers compared with non‐stone formers. The investigators conclude that an association does exist between urine oxalate and citrate in humans, such that higher oxalate excretion associates with higher urine citrate excretion, and that the magnitude of this association is attenuated in stone formers so that they are at higher risk for calcium oxalate stones.

Is there a signal that supports the proposed hypothesis? It is unclear. The conclusions are limited for a number of reasons, some of which are acknowledged by the investigators. The study was designed to show an association between urine oxalate and citrate excretion but not to prove that this association can be explained by the proposed mechanism between SLC2646 and NaDC1. Many exogenous (diet composition or eating behavior) or endogenous (obesity, chronic kidney disease, or acidosis) factors can independently affect urine oxalate and citrate excretion. The investigators attempt to control for some of these factors, but they do not report relevant factors that may affect this relationship such as age (for the non‐stone forming population), weight, serum chemistries, or kidney function. Obesity is associated with higher urine oxalate excretion (Amin et al., 2018; Sakhaee, 2018), and acidosis will decrease urine citrate excretion (Alpern, 1995). Increasing age may be associated with several factors that decrease urine citrate excretion, including steady‐state acid accumulation (Frassetto & Sebastian, 1996) and worsening kidney function (Gianella et al., 2021; Goraya et al., 2019). Finally, the dietary habits of subjects could be responsible for the positive correlation that was observed between urine oxalate and citrate excretion: people may tend to ingest foods that are rich in both oxalate and alkali. Moreover, it may be possible that stone formers ingest a diet that contains more oxalate than alkali because they make different dietary choices. A more definitive test of the proposed mechanism would be to feed stone formers graded amounts of oxalate with a controlled diet and then measure ensuing urine citrate excretion.

In conclusion, the simplicity of the original hypothesis by Prochaska et al. belies the complexity of the physiology underlying oxalate and citrate homeostasis in humans. The small intestine, liver, and proximal tubule all contribute to oxalate excretion, while the status of acid–base balance plays a significant factor in regulating citrate excretion. Without implementation of carefully controlled studies that account for age, sex, demographics, co‐morbid illness, and diet, it is unlikely that extrapolation of the interaction between SLC2646 and NaDC1 at the cellular level will lead to an accurate ab initio prediction of how the body integrates the control of oxalate and citrate excretion. Nonetheless, the investigators should be commended for testing whether a specific biological/cellular mechanism underlying urine citrate and oxalate handling exists in a human clinical population. Working from first principles, the investigators have taken the necessary first step.
